# Fasting and postprandial trimethylamine *N*‐oxide in sedentary and endurance‐trained males following a short‐term high‐fat diet

**DOI:** 10.14814/phy2.14970

**Published:** 2021-08-17

**Authors:** Cortney N. Steele, Mary Elizabeth Baugh, Laura E. Griffin, Andrew P. Neilson, Brenda M. Davy, Matthew W. Hulver, Kevin P. Davy

**Affiliations:** ^1^ Division of Renal Diseases and Hypertension University of Colorado Anschutz Medical Aurora CO USA; ^2^ Center for Transformative Research on Health Behaviors Fralin Biomedical Research Institute at Virginia Tech Carilion Roanoke VA USA; ^3^ Department of Food, Bioprocessing and Nutrition Sciences North Carolina State University Kannapolis NC USA; ^4^ Plants for Human Health Institute Kannapolis NC USA; ^5^ Department of Human Nutrition, Foods, and Exercise Virginia Tech Blacksburg VA USA; ^6^ Translational Obesity Research Interdisciplinary Graduate Education Program Virginia Tech Blacksburg VA USA

**Keywords:** endurance‐trained, high‐fat diet, sedentary, trimethylamine *N*‐oxide

## Abstract

Gut bacteria release trimethylamine (TMA) from dietary substrates. TMA is absorbed and is subsequently oxidized in the liver to produce trimethylamine *N*‐oxide (TMAO). Plasma TMAO levels are positively correlated with risk for type 2 diabetes (T2D) and cardiovascular disease (CVD). High‐fat diet (HFD) consumption has been reported to increase fasting and postprandial TMAO in sedentary individuals. However, whether the increase in TMAO with consumption of an HFD is observed in endurance‐trained males is unknown. Healthy, sedentary (*n* = 17), and endurance‐trained (*n* = 7) males consumed a 10‐day eucaloric diet comprised of 55% carbohydrate, 30% total fat, and <10% saturated fat prior to baseline testing. Blood samples were obtained in a fasted state and for a 4‐hour high‐fat challenge (HFC) meal at baseline and then again following 5‐day HFD (30% carbohydrate, 55% total fat, and 25% saturated fat). Plasma TMAO and TMA‐moiety (choline, betaine, L‐carnitine) concentrations were measured using isocratic ultraperformance liquid chromatography‐tandem mass spectrometry. Age (23 ±3 vs. 22 ± 2 years) and body mass index (23.0 ± 3.0 vs. 23.5 ± 2.1 kg/m^2^) were similar (both *p* > 0.05) in the sedentary and endurance‐trained group, respectively. *V*O_2max_ was significantly higher in the endurance‐trained compared with sedentary males (56.7 ± 8.2 vs. 39.9 ± 6.0 ml/kg/min). Neither the HFC nor the HFD evoked a detectable change in plasma TMAO (*p* > 0.05) in either group. Future studies are needed to identify the effects of endurance training on TMAO production.

## Graphical abstract

Plasma trimethylamine *N*‐oxide (TMAO) levels are positively correlated with risk for cardiovascular disease and consumption of a high‐fat diet has been reported to increase TMAO. Healthy, sedentary (*n* = 17), and endurance‐trained (*n* = 7) males consumed a 10‐day eucaloric diet and a 5‐day high‐fat diet. There were no significant differences in fasting (*p* > 0.05) or postprandial TMAO (*p* > 0.05) between sedentary and endurance‐trained individuals before or following the high‐fat diet, despite the endurance‐trained group consuming more dietary precursors.

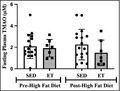

## 1 INTRODUCTION

Cardiovascular disease (CVD) remains the leading cause of death and disability in the United States (Mensah & Brown, 2007). Recently, a link has been reported between the gut microbial metabolism of trimethylamine (TMA) moieties from dietary sources (choline, phosphatidylcholine, L‐carnitine, etc.) and CVD (Liu & Dai, 2020; Tang & Hazen, 2017; Tang et al., 2013; Wang et al., 2011).

Phosphatidylcholine, the major phospholipid in cell membranes, is abundant in the typical western diet and is a major source of choline in the diet of omnivores (Tang & Hazen, 2017; Tang et al., 2013). Gut microbes metabolize dietary choline to release TMA primarily via the action of the enzyme *cutC* TMA lyase. TMA is absorbed into the circulation and then oxidized by hepatic flavin monooxygenase 3 (FMO3) to produce TMAO (Romano et al., 2015). The ingestion of foods containing TMA moieties including choline, betaine, and L‐carnitine results in a significant rise in plasma TMAO concentrations (Taesuwan et al., 2017; Tang & Hazen, 2017). As such, the diet has been implicated as playing a key role in the generation of TMAO.

Boutagy and colleagues (Boutagy et al., 2015b) previously reported a significant increase in 4 h postprandial TMAO in young healthy males following an isocaloric, high‐ fat diet (HFD; 55% fat) for 5 days. Fasting TMAO increased after consuming a hypercaloric, HFD (55% fat) for 4 weeks (Boutagy et al., 2015a). Overall, HFD appear to be associated with increased fasting and postprandial TMAO in sedentary to recreationally trained individuals (Boutagy et al., 2015a, 2015b; Park et al., 2019). However, whether endurance‐trained individuals respond similarly to an HFD and whether TMAO is reduced in endurance‐trained individuals remain unclear. Lifestyle modifications that include both caloric restriction and exercise have been reported to reduce TMAO levels in humans (Erickson et al., 2019) and rodents (Zhang et al., 2017). Previous studies suggest that exercise training can alter the composition of the gut microbiome (Mach & Fuster‐Botella, 2017; Mailing et al., 2019; Mitchell et al., 2019) which, in turn, could influence TMAO production in endurance‐trained individuals. The purpose of this investigation was to test the hypothesis that fasting and postprandial TMAO concentrations are reduced in endurance‐trained males compared with sedentary males before and following a HFD. Our sample was limited to that included in the parent study which focused on skeletal muscle metabolism (Baugh et al., 2020; Bowser et al., 2020).

## 2 MATERIALS AND METHODS

### 2.1 Participants

Seventeen healthy, sedentary (<2 h of physical activity/week for the previous 3 months and no exercise regime), and seven endurance‐trained (running for ≥5 h/week and competed in ≥2 races of ≥10 kilometers over the last 12 months) males aged 18–40 years met the inclusion criteria and subsequently completed the study (Table 1). Individuals were excluded if they were taking antibiotics, prebiotics, or probiotics 3 months prior to screening. The protocol was approved by the Virginia Polytechnic Institute and State University Institutional Review Board (#06‐367) and both written and verbal informed consent were obtained from each participant. The present study was a secondary analysis that relied on stored blood samples collected from a study in which the experimental design and procedures have been described previously (Baugh et al., 2020; Bowser et al., 2020). Therefore, our sample size was limited to males in our previous study focused on skeletal muscle metabolism because in our experience females have declined to undergo the biopsy procedures (Baugh et al., 2020; Bowser et al., 2020).

**TABLE 1:** Inclusion & exclusion criteria

Inclusion criteria	Exclusion criteria
Males age 18–40 years	BMI > 30 kg/m^2^
Weight stable for previous 6 months (±2.5 kg)	Fasting Blood glucose >126 mg/dl
Sedentary‐to‐recreationally active or endurance trained	TCHOL >240 mg/dL; LDL‐C > 130 mg/dl
Habitual dietary fat intake ≤40%	BP > 140/90 mmHg
Non‐smokers	Taking any medications or supplements known to influence these blood markers or study outcome variables
No history of CVD	Food allergies or lactose intolerance

Abbreviations: BMI, body mass index; BP, blood pressure; CVD, cardiovascular disease; LDL‐C, low‐density lipoprotein‐cholesterol; TCHOL, total cholesterol.

### 2.2 Experimental design

**FIGURE 1:**
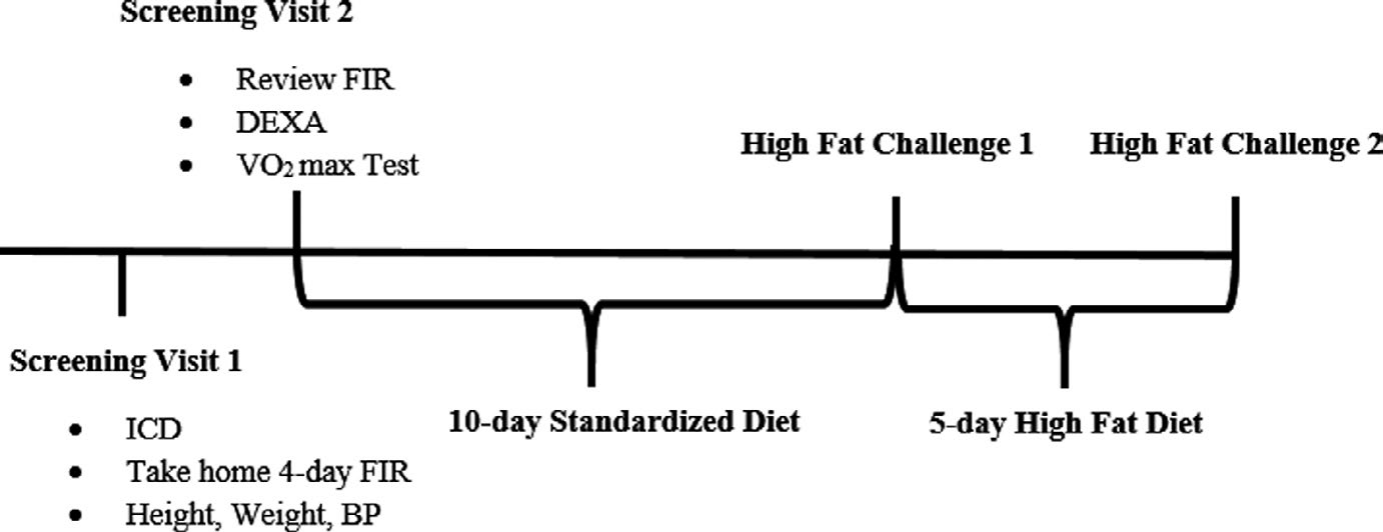
Study timeline

Individuals completed two screening visits (Figure 1) to evaluate whether all inclusion criteria were met. During the initial screening visit, participants completed a fasting blood draw and anthropometric measures. They were also given instructions to record habitual dietary intake with 4‐day food intake records (3 weekdays and 1 weekend day). During the second visit, participants reviewed their 4‐day food intake records with a registered dietitian. Body weight was measured with a digital scale and height was measured using a stadiometer (Model 5002, Scale‐Tronix, Inc.). Brachial arterial pressure was measured in a seated position using automated sphygmomanometry (Press‐Mate BP 8800, Colin Medical Instruments Corp, San Antonio, TX, USA). Body composition was assessed via dual x‐ray absorptiometry (DXA, Lunar iDXA with enCORE version 17 software, GE Healthcare). Maximal oxygen consumption (*V*O_2max_) was measured during incremental treadmill exercise to volitional fatigue using indirect calorimetry (ParvoMedics TrueOne 2400, Sandy, UT). Plasma glucose was measured via a YSI 2300 Stat Plus glucose analyzer (Yellow Springs Instruments, Yellow Springs, OH). Lipid and lipoprotein concentrations were measured in a certified commercial laboratory (Solstas Lab Partners, Roanoke, VA).

### 2.3 Controlled feeding and physical activity

All diets were controlled and standardized to reduce the individual variability of habitual diet intake on study outcomes. The Mifflin‐St. Jeor equation was used to determine energy requirements determined from age, weight, height, and sex (Mifflin et al., 1990). Participants consumed a eucaloric (i.e., weight maintenance) standardized diet (55% carbohydrate, 30% total fat, <10% saturated fat) for 10 days. Following the isocaloric diet, participants completed a high‐fat challenge meal testing session consisting of two sausage, egg, and cheese breakfast sandwiches (820 kcals; 63% fat [21% saturated fat], 25% carbohydrate. 317.4 mg choline, 62.5 mg betaine, 8.1 mg L‐carnitine). Participants then completed a eucaloric 5‐day HFD phase (30% carbohydrate, 55% total fat, 25% saturated fat) and a second high‐fat challenge meal testing session. The Virginia Tech Metabolic Kitchen in the Laboratory for Eating Behaviors and Weight Management provided all foods and beverages to the participants. Food and beverage items were prepped and weighed using a digital benchtop scale (Practum 5101‐1S, Sartorius; Goettingen, Germany) to meet the requirements of the planned menu in grams (g) within ±0.9 g. Participants ate a supervised breakfast daily in the dining laboratory and were then given a cooler with food and beverages for the remainder of the day. During feeding periods, participants were given all foods and beverages with the exception of water. Participants were asked to consume all food in the cooler with optional snack modules if needed, which matched the macronutrient content of the controlled diet (~250 kcals). Body weight was measured on a digital scale (Scale‐Tronix Model 5002, Welch Allyn, Skaneateles Falls, NY, USA) during each visit to the dining laboratory. Participants remained weight stable throughout the study. If body weight change was >1.36 kg, the energy intake was adjusted accordingly to maintain weight stability. Short‐term (i.e., days to weeks) fluctuations in body weight in individuals not intending to gain or lose body weight may be as large ±3 kg (Bhutani et al., 2017). We selected a more conservative threshold of 1.36 kg (3 lbs). Participants were instructed to not change their exercise/physical activity levels throughout the study with the exception of avoiding moderate to vigorous physical activity for 24 h before testing sessions.

### 2.4 Procedures and blood collection

Daily choline and betaine intake were assessed for habitual, standardized, and high‐fat diets via Nutrition Data System for Research software (NDS‐R v. 2014; University of Minnesota, Minneapolis, MN, USA). Daily L‐carnitine intake was calculated using the food group classification and serving amount in NDS‐R, converting these servings into grams. The gram servings of each food were then converted to milligram intake of L‐carnitine, as previously reported (Baugh et al., 2018; Boutagy et al., 2015a, 2015b; Demarquoy et al., 2004). All measurements and testing took place between the hours of 5:00 and 11:00 a.m. in the Human Integrative Physiology Laboratory. Participants came to the laboratory after an overnight fast (~12 h) and avoided vigorous physical activity for 36 h. Blood was obtained in a fasted state and each hour for 4 h after the consumption of the challenge meal. Blood samples were centrifuged and plasma was stored at −80°C until analysis could occur.

### 2.5 Mass spectrometry

Plasma TMAO, choline, betaine, and L‐carnitine were assessed using isocratic ultraperformance liquid chromatography‐tandem mass spectrometry as described previously (Boutagy et al., 2015a, 2015b). Prior to analysis, samples were brought to room temperature. The internal standard/extraction solution was prepared by diluting 1 ml of concentrated stock solution [aqueous choline chloride‐d_9_ (25 μM, Sigma, St. Louis, MO), betaine‐d_9_, TMAO‐d_9_ (25 μM, Cambridge Isotope Laboratories, Tewksbury, MA), and L‐carnitine‐d_9_ (120 μM, Cambridge Isotope Laboratories)] to a final volume of 100 ml with acetonitrile (ACN). Plasma (25 μl) and internal standard/extraction solution (300 μl) were combined, vortexed, and centrifuged. (17,000*g*, 3 min, room temperature). Supernatants were syringe filtered (PTFE, 4 mm, 0.2 μm pore size) into certified Waters LC‐MS vials w/with spring‐loaded deactivated glass inserts (150 μl) and analyzed immediately. Samples were analyzed (5 μl) on a Waters ACQUITY UPLC‐MS/MS instrument (Milford, MA). Separations were performed on a Waters BEH HILIC column (2.1 × 100 mm; 1.7 μm particle size) with a BEH HILIC VanGuard pre‐column (2.1 × 5 mm; 1.7 μm). Column and sample temperatures were 30 and 10°C, respectively. The mobile phases were 15 mM ammonium formate, pH 3.5 (phase A), and ACN (phase B). The flow rate was 0.65 ml/min and isocratic elution was achieved (20% A/80% B) over 3 min. Following separation, analytes and internal standards (IS) were quantified using electrospray ionization (ESI) in (+)‐mode. Source and capillary temperatures were 150 and 400°C, respectively. Capillary voltage was 0.60 kV and desolvation and cone gas (both N_2_) flow rates were 800 and 20 L/h, respectively. Collision‐induced dissociation was performed using Ar as the collision gas. Compounds were quantified using optimized multi‐reaction monitoring (MRM) functions (Table 2). MRMs were optimized to achieve 12 points/10 s peak and the detection span was ±0.2 amu. Quantification was performed using the ratio of the target analyte and respective IS peak areas based on authentic external standard curves prepared using a wide range of target analyte concentrations (choline chloride, TMAO, betaine, and L‐carnitine, [Sigma, St. Louis, MO, USA]) bracketing the peak areas observed in the plasma samples and the same IS concentrations used to prepare the plasma samples.

**TABLE 2:** MRM settings for UPLC‐MS/MS detection

Compound	Retention time (min)	MW (g/mol)	Parent [M+H]^+^ (m/z)	Daughter (m/z)	Cone voltage (V)	Collision energy (eV)
Betaine	1.25	117.15	118.24	59.42	44	18
Betaine‐d_9_	1.25	126.14	127.3	68.10	46	18
Choline	1.13	103.16	104.2	60.02	38	16
Choline‐d_9_	1.11	112.16	113.32	69.08	40	16
TMAO	2.01	75.11	76.16	58.91	40	10
TMAO‐d_9_	1.98	84.12	85.22	68.1	40	12
L‐Carnitine	2.09	161.20	162.26	84.99	34	20
L‐Carnitine‐d_9_	2.08	170.25	171.28	84.99	34	20

### 2.6 Statistical analysis

Statistical analyses were conducted using SPSS statistical software (version 24, 2016; IBM, Armonk, NY, USA). Prism (GraphPad Software, version 7.03 for Windows, 2018, La Jolla, CA, USA) was used to generate figures and calculate integrated areas under the curve (iAUC). Normality testing was performed using Kolmogorov–Smirnov tests. Variables that were not normally distributed were log‐transformed prior to further statistical analysis. Independent samples *t*‐tests were used to compare group baseline characteristics and habitual dietary variables. Two‐way repeated‐measure analysis of variance was used to assess the effects of group, diet (standardized vs. HFD), and group × diet interaction on dietary variables. Two‐way repeated‐measures analysis of variance was used to assess the effects of group, HFD intervention (pre vs. post), and group × HFD intervention on dependent variables. The significance level of *p* < 0.05 was set *a priori* for all statistical tests.

## 3 RESULTS

### 3.1 Participant characteristics

Baseline participant characteristics (Table 3) including age, body mass index, percent body fat, body weight, HDL‐cholesterol, triglycerides, and fasting plasma glucose did not differ between sedentary and endurance‐trained males (all *p* > 0.05). Total cholesterol (*p* = 0.005) and LDL‐cholesterol (*p* = 0.003) were lower in the endurance‐trained compared with sedentary males. *V*O_2max_ was higher (*p* = 0.005) in the endurance‐trained group compared with the sedentary group. Body weight and percent body fat did not change during the intervention in either group (all *p* > 0.05).

**TABLE 3:** Participant characteristics at baseline

	Sedentary (*n* = 17)	Endurance‐trained (*n* = 7)
Age (years)	23 ± 3	22 ± 2
BMI (kg/m^2^)	23.0 ± 3.0	23.5 ± 2.1
Body fat (%)	21.0 ± 5.0^a^	15.8 ± 5.0
FFM (kg)	18.7 ± 2.1	20.8 ± 2.9
Body weight (kg)	73.5 ± 11.1	74.0 ± 12.0
Fasting plasma glucose (mg/dl)	88 ± 10^b^	82 ± 17
Plasma total cholesterol (mg/dl)	185 ± 27	150 ± 16*
Plasma LDL Cholesterol (mg/dl)	111 ± 28	73 ± 19*
Plasma HDL Cholesterol (mg/dl)	54 ± 13	63 ± 10
Plasma triglycerides (mg/dl)	99 ± 36	68 ± 21
*V*O_2max_ (ml/kg/min)	39.9 ± 6.0^c^	56.7 ± 8.2*
*V*O_2max_/FFM, (ml/min)	1.95 ± 0.4	2.74 ± 0.3*	

Data are presented as means ± SD.

Abbreviations: BMI, body mass index; FFM, fat‐free mass; HDL, high‐density lipoprotein‐cholesterol; LDL, low‐density lipoprotein‐cholesterol.

^a^ *n* = 16 for the group.

^b^ *n* = 13 for the group.

^c^ *n* = 4 for the group.

* *p* < 0.05 endurance‐trained versus sedentary group, independent *t*‐test.

### 3.2 Dietary intake

Habitual percent fat intake was lower (*p* = 0.005) in the endurance‐trained group compared with the sedentary (Table 4). The endurance‐trained group consumed more habitual carbohydrates grams (*p* = 0.006) and percent carbohydrate (*p* = 0.01) compared with the sedentary group (Table 4). Habitual total fiber (*p* = 0.0002), soluble fiber (*p* = 0.004), and insoluble fiber (*p* = 0.0005) were also higher in the endurance‐trained group compared with sedentary. Habitual TMAO moiety (betaine, choline, L‐carnitine) intake, regardless of how expressed, was similar between groups (All *p* > 0.05). Standardized and high‐fat dietary intakes were significantly higher in the endurance‐trained males (Table 5).

**TABLE 4:** Habitual dietary intake of sedentary and endurance‐trained participants

	Sedentary (*n* = 17)	Endurance trained (*n* = 7)
Energy (kcals/d)	2311 ± 359	2607 ± 791
Fat (g/d)	93 ± 20	88 ± 41
Fat intake (%)	36 ± 4	29 ± 8*
Carbohydrate (g/d)	261 ± 47	344 ± 88*
Carbohydrate intake (%)	45 ± 6	54 ± 10*
Protein (g/d)	93 ± 21	103 ± 37
Protein intake (%)	16 ± 4	16 ± 4
Betaine (mg/d)	158 ± 36	286 ± 72
Choline (mg/d)	370 ± 88	449 ± 72
L‐Carnitine (mg/d)	63 ± 24	40 ± 7
Total fiber (g/d)	16 ± 5	30 ± 10*
Soluble fiber (g/d)	5 ± 1	9 ± 4*
Insoluble fiber (g/d)	11 ± 3	21 ± 9*

Data are presented as means ± SD.

* *p* < 0.05 endurance‐trained versus sedentary group, independent *t*‐test.

**TABLE 5:** Controlled feeding dietary intake of sedentary and endurance‐trained participants

	Sedentary (*n* = 17)		Endurance trained (*n* = 7)		*p*‐values		
	Standardized diet	High‐fat diet	Standardized Diet	High‐fat diet	Group	Diet	Group × diet
Energy (kcals/d)	2714 ± 259	2742 ± 319	3438 ± 486	3729 ± 590	<0.001	0.189	0.277
Total fat (g/d)	91 ± 8	168 ± 19	119 ± 7	227 ± 36	<0.001	<0.001	0.015
Polyunsaturated fatty acids (g/d)	25 ± 3	32 ± 4	31 ± 4	46 ± 9	<0.001	<0.001	0.007
Monounsaturated fatty acids (g/d)	33 ± 3	51 ± 6	43 ± 6	66 ± 10	<0.001	<0.001	0.207
Saturated fat (g/d)	26 ± 3	75 ± 9	35 ± 6	100 ± 16	<0.001	<0.001	0.004
Carbohydrate (g/d)	377 ± 36	207 ± 24	471 ± 65	288 ± 46	<0.001	<0.001	0.577
Protein (g/d)	103 ± 10	101 ± 11	132 ± 19	136 ± 21	<0.001	0.811	0.455
Betaine (mg/d)	310 ± 13	202 ± 6	362 ± 16	238 ± 15	0.002	<0.001	0.545
Choline (mg/d)	325 ± 8	257 ± 15	410 ± 18	336 ± 21	<0.001	<0.001	0.768
L‐Carnitine (mg/d)	51 ± 2	54 ± 2	63 ± 3	67 ± 4	<0.001	0.218	0.822
Total fiber (g/d)	18 ± 2	13 ± 2	22 ± 3	17 ± 3	<0.001	<0.001	0.589
Soluble fiber (g/d)	7 ± 1	5 ± 1	8 ± 1	7 ± 1	<0.001	<0.001	0.944
Insoluble fiber (g/d)	11 ± 1	8 ± 2	14 ± 2	10 ± 2	<0.001	<0.001	0.373

Data are presented as means ± SD.

Group, endurance‐trained versus sedentary.

Diet, high‐fat diet versus standardized diet.

### 3.3 Fasting and postprandial plasma TMAO and TMA moiety concentrations

**FIGURE 2:**
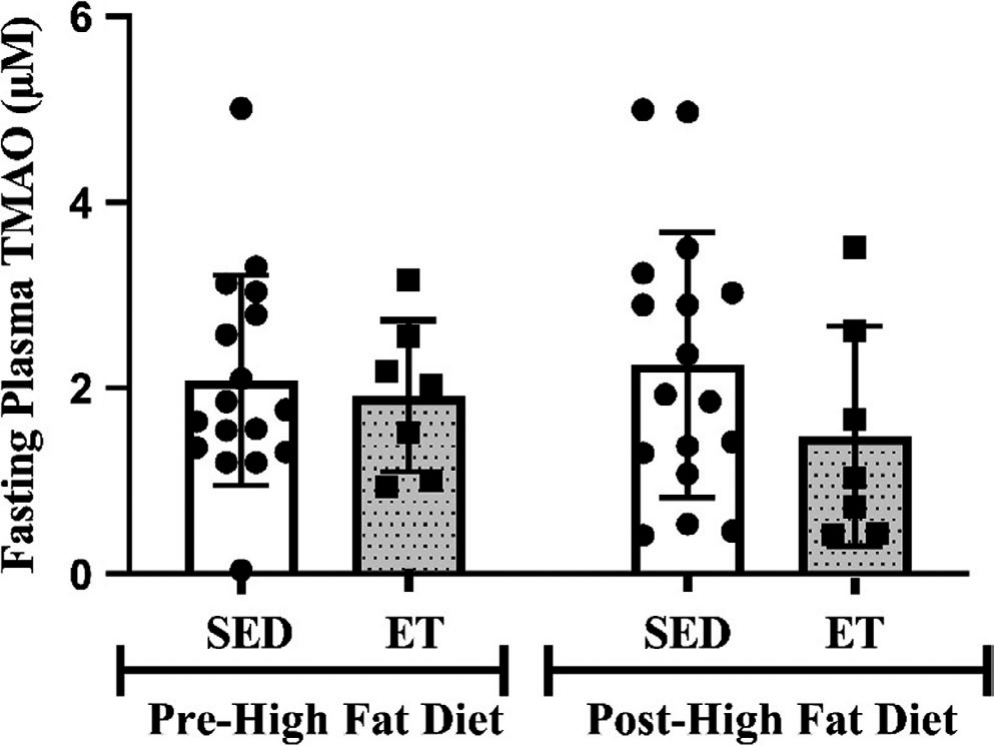
Fasting plasma TMAO concentration in SED and ET before and followeing the high‐fat diet. Values are expressed as mean ± SD. ET, endurance‐trained group; SED, sedentary group; TMAO, trimethylamine *N‐*oxide

**FIGURE 3:**
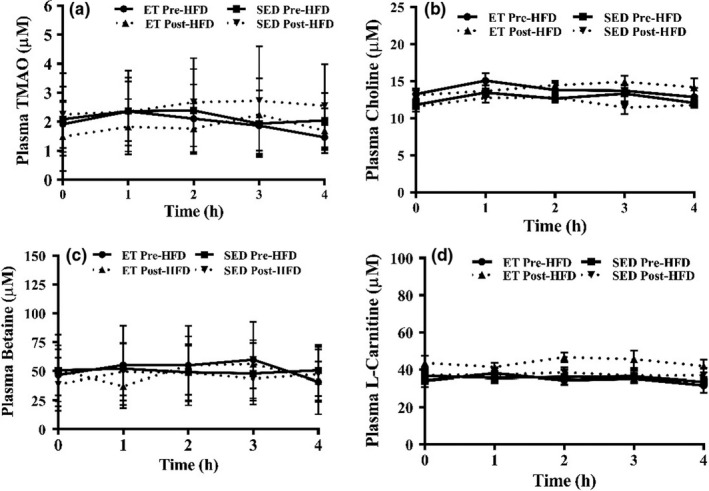
Fasting and postprandial plasma TMAO (panel a), Choline (panel b), Betaine (panel c), L‐carnitine (panel d) baseline and post‐ high fat diet. Values are expressed as mean ± SD. ET, endurance‐trained group; TMAO, trimethylamine *N‐*oxide; HFD, high fat diet; SED, sedentary group

Fasting plasma TMAO concentrations between the groups were similar at baseline (*p* = 0.724) (Figure 2). Fasting and postprandial TMAO concentration did not change in the endurance‐trained or sedentary groups with the intervention (*p* > 0.05) (Figure 3). There were no differences in fasting concentrations of choline, betaine, and L‐carnitine (all *p* > 0.05) between endurance‐trained or sedentary groups at baseline or in response to the intervention (Figure 3). The iAUC for TMAO did not significantly change with the diet in either group (*p* > 0.05). The iAUC for plasma choline was higher from 0 to 4 h in endurance‐trained males post‐high‐fat diet (56.7 ± 7.2 vs. 48.7 ± 8.3 µM/h, *p* = 0.032) when compared to sedentary males. There were no significant correlations between *V*O_2max_ and TMAO or its moieties.

## 4 DISCUSSION

The major new finding of the present investigation is that neither fasting nor postprandial plasma TMAO concentrations differed between endurance‐trained and sedentary males that were otherwise similar in their baseline characteristics with the exception of VO_2 max_ and blood cholesterol concentrations (Table 3). In addition, neither fasting nor postprandial TMAO concentrations changed following the HFD in either group despite significantly higher dietary intake of TMA precursors in the endurance‐trained males.

The results of previous studies suggest that postprandial, but not fasting, TMAO increases in healthy young males following 5 days of consuming an isocaloric, HFD (Boutagy et al., 2015a). However, fasting TMAO was also reported to increase following a 4‐week hypercaloric, HFD in a similar population (Boutagy et al., 2015b). Taken together with the findings of the present study, these studies suggest that the consumption of HFD for a sufficiently longer period of time might be necessary to increase fasting and postprandial TMAO.

We found that TMAO was similar in endurance‐trained and sedentary participants at baseline and following the HFD despite higher intake of TMA precursors in the former. The reasons for this remain uncertain but whether the higher intake of TMA‐moieties during the HFD in the endurance‐trained group was of sufficient magnitude to impact plasma TMAO concentrations is unclear. As such, there may be differences in the composition of the gut microbiota and/or hepatic FMO3 activity in the endurance‐trained compared with sedentary individuals which could have influenced TMAO production. In this regard, there is accumulating evidence that exercise training may alter gut microbiota composition (Mach & Fuster‐Botella, 2017; Mailing et al., 2019; Mitchell et al., 2019; Zhao et al., 2018). However, to our knowledge, there is currently no information available on whether the abundance of TMA‐releasing bacteria or the expression of *CutC* TMA lyase are reduced in endurance‐trained athletes. Similarly, whether FMO3 activity is modified by endurance training is unknown.

Erickson et al. (Erickson et al., 2019) recently reported that only percentage change (not the absolute level or change) in fasting TMAO concentration was reduced following 12 weeks of a hypocaloric diet plus exercise but not an isocaloric diet plus exercise. In addition, baseline fasting TMAO was correlated with baseline *V*O_2max_ (r = 0.67, *p* = 0.004). Our observation of similar fasting TMAO (absolute values) in the endurance‐trained and sedentary males in the present study would appear to be inconsistent with this prior observation. The reasons for this discrepancy are unclear but differences in how TMAO values were expressed and the study population might contribute. In addition, it is important to emphasize that Erickson and colleagues (Erickson et al. 2019) included sedentary older adults with obesity in their study and the independent role of exercise in contributing to the reduction in plasma TMAO remains unclear. It is also important to note that individuals in the current investigation remained weight stable whereas, in the study conducted by (Erickson et al., 2019), participants lost a significant amount of subcutaneous abdominal fat following the intervention, which may independently decrease TMAO production.

There are several strengths of this investigation. First, we utilized a rigorous controlled feeding design, in which all foods and beverages were provided to participants during both standardized and HFD phases. Second, each participant's weight was measured daily, and when necessary, adjustments in energy intake were made to ensure that weight stability was maintained. Finally, differences in self‐reported exercise were used to determine endurance training status and verified using laboratory‐based assessments of maximal oxygen consumption.

There are some limitations of our study that should be addressed. First, the study relied on stored samples from a prior study to test the stated hypothesis. As such, it is unclear whether implementing diets matched for TMA precursor intake would result in a different outcome. Second, the sample size of the present study was small and limited to males aged 18–40 years, thus limiting the generalizability of the findings. Third, the diet duration was relatively short; it is possible that a longer dietary intervention could produce different findings. However, the composition of the gut microbiome can be altered in as few as 1 to 2 days on a species and family level (Sonnenburg & Bäckhed, 2016). Therefore, it is reasonable to believe that the length of our intervention would be sufficient if changes in gut microbiota occurred leading to altered TMA production. Fourth, the differences in *V*O_2max_ between the sedentary and endurance‐trained males in the present study were relatively modest. As such, whether the inclusion of groups with larger differences in *V*O_2max_ would result in different findings is unknown. Fifth, we did not document compliance ensuring participants maintained their physical activity levels. This remains a limitation. Finally, we did not quantify either gut microbiota composition or hepatic FMO3 expression in the present study; the latter could not be done with liver biopsies. As such, the mechanism into why TMAO was similar in the two groups following the HFD despite higher intake of TMA precursors in the endurance‐trained males remains unclear.

## 5 CONCLUSION

In summary, our findings suggest that neither fasting nor postprandial TMAO concentrations changed significantly following the HFD in the endurance‐trained group compared with sedentary males despite higher consumption of TMA dietary substrates in the endurance‐trained group. Future studies are needed to explore mechanisms in which TMAO can be altered by exercise training in relation to TMA precursor intake.

## DISCLOSURES

No conflicts of interest, financial or otherwise are declared by the authors.

**AUTHOR CONTRIBUTIONS**

Conceptualization, M.W.H., B.M.D., and K.P.D.; Methodology, M.W.H. A.P.N., B.M.D., and K.P.D.; Formal Analysis, C.N.S., L.E.G., A.P.N., and K.P.D.; Data Curation, C.N.S. and M.E.B.; Writing—Original Draft Preparation, C.N.S. and K.P.D.; Writing—Reviewing and Editing, C.N.S., M.E.B., L.E.G., A.P.N., B.M.D., M.W.H., and K.P.D.
